# Spectrum of COVID-19 cases in Arkhangelsk, Northwest Russia: Findings from a population-based study linking serosurvey, registry data, and self-reports of symptoms

**DOI:** 10.1371/journal.pone.0311287

**Published:** 2024-10-11

**Authors:** Ekaterina Krieger, Alexander V. Kudryavtsev, Ekaterina Sharashova, Olga Samodova, Anna Kontsevaya, Vitaly A. Postoev

**Affiliations:** 1 Department of Community Medicine, UiT The Arctic University of Norway, Tromsø, Norway; 2 International Research Competence Centre, Northern State Medical University, Arkhangelsk, Russian Federation; 3 Department of Infectious diseases, Northern State Medical University, Arkhangelsk, Russian Federation; 4 Department of Public Health, National Medical Research Centre for Therapy and Preventive Medicine, Moscow, Russian Federation; 5 Department of Research Methodology, Northern State Medical University, Arkhangelsk, Russian Federation; PLOS ONE, UNITED KINGDOM OF GREAT BRITAIN AND NORTHERN IRELAND

## Abstract

**Introduction:**

The spectrum of COVID-19 manifestations makes it challenging to estimate the exact proportion of people who had the infection in a population, with the proportion of asymptomatic cases likely being underestimated. We aimed to assess and describe the spectrum of COVID-19 cases in a sample of adult population aged 40–74 years in Arkhangelsk, Northwest Russia, a year after the start of the pandemic.

**Materials and methods:**

A population-based survey conducted between February 24, 2021 and June 30, 2021 with an unvaccinated sample aged 40–74 years (N = 1089) combined a serological survey data, national COVID-19 case registry, and self-reported data on COVID-19 experience and symptoms. Based on the agreement between these sources, we classified the study participants as non-infected and previously infected (asymptomatic, non-hospitalized and hospitalized symptomatic) cases, and compared these groups regarding demographics, lifestyle and health characteristics.

**Results:**

After a year of the pandemic in Arkhangelsk, 59.7% 95% confidence intervals (CI) (56.7; 62.6) of the surveyed population had had COVID-19. Among those who had been infected, symptomatic cases comprised 47.1% 95% CI (43.2; 51.0), with 8.6% 95% CI (6.6; 11.1) of them having been hospitalized. Of the asymptomatic cases, 96.2% were not captured by the healthcare system. Older age was positively associated, while smoking showed a negative association with symptomatic COVID-19. Individuals older than 65 years, and those with poor self-rated health were more likely to be hospitalized.

**Conclusion:**

More than half of the infected individuals were not captured by the healthcare-based registry, mainly those with asymptomatic infections. COVID-19 severity was positively associated with older age and poor self-rated health, and inversely associated with smoking. Combining different sources of surveillance data could reduce the number of unidentified asymptomatic cases and enhance surveillance for emerging infections.

## Introduction

The novel coronavirus disease 2019 (COVID-19) caused by the severe acute respiratory syndrome coronavirus-2 (SARS-CoV-2) is a highly contagious disease, which spread around the world despite the infection control efforts. Infected individuals may develop a range of COVID-19 manifestations, from asymptomatic or mild to severe disease and death. This range of the disease severity is termed as the spectrum of COVID-19 [1]. The COVID-19 severity depends on the SARS-CoV-2 variant, the viral load, and host factors such as sex, age, and chronic diseases [2–4].

The variety of individual responses to the infection makes it challenging to determine the exact proportion of the infected people in a population. Based on the iceberg concept of infectious diseases, detected COVID-19 cases represent only the emerging tip of the iceberg, while asymptomatic cases comprise the invisible base [5].

When the coronavirus pandemic was declared, most countries implemented non-pharmaceutical interventions to prevent further disease transmission [6]. During the initial stage of the pandemic in Russia, nationwide paid non-working days were established [7–9]. Subsequently, regional governments implemented and updated non-pharmaceutical interventions based on the local epidemiological situation, in accordance with Presidential Decree #316, dated May 11, 2020 [10]. These measures generally aligned with the recommendations of the Russian Federal Service for Surveillance on Consumer Rights Protection and Human Wellbeing (Rospotrebnadzor) and included mandatory wearing facemasks in public settings or transport, travel restrictions, limitations on commercial activities, and the closure of educational institutions and non-essential services [11]. Individuals aged 65 years and older, as well as those with chronic conditions such as diabetes, respiratory and cardiovascular issues, chronic kidney diseases, neoplasms in any location, organ and tissue transplant recipients, and pregnant women, were advised to self-isolate [11]. Other COVID-19 non-pharmaceutical interventions, implemented in Russia as well as worldwide, included testing strategies, contact tracing, and isolation of infected people [6].

The Polymerase Chain Reaction (PCR) test was used to detect ongoing infections by capturing SARS-CoV-2 genetic material in nasopharyngeal swabs [12, 13]. Since infected individuals may transmit SARS-CoV-2 regardless of their symptomatic status, preventing the transmission to the susceptible population depends on a number of individuals with the ongoing infection who remained unidentified [14, 15]. The detection proportion refers to the number of positive tests among all tests done and reflects testing strategy effectiveness. During the first six months of the pandemic, the global detection proportion was estimated to be 9.8% (range 1.2–66.8%) [16]. The highest detection proportions were in Australia (66.8%) and Iceland (60.3%). In Russia, the detection proportion was estimated to be 25.4% [16].

The data about all diagnosed COVID-19 cases and COVID-19 vaccine recipients were accumulated in the Federal Registry of COVID-19 Patients (later in this paper referred to as the COVID-19 case registry or the case registry) and the Federal Registry of the Vaccinated Against COVID-19 (later in this paper referred to as the vaccination registry) respectively, as regulated by Russian Government Decree #373 dated March 31, 2020 [17]. The registries gathered COVID-19-related data from patients’ electronic health records contained by information systems of governmental non-military health services. The data accumulated in the case registry were based on positive results of the PCR tests, rather than clinical symptoms [12, 18]. Patients with severe disease were more likely to be PCR-tested, while asymptomatic cases and those with minor symptoms might have failed to seek medical advice or undergo testing [19]. Due to a limited capacity of PCR testing and the high load on the healthcare system at the earlier stages of the pandemic, testing was restricted to patients with signs of pneumonia and individuals with other respiratory symptoms in the higher risk groups (healthcare workers, close contacts of a confirmed case, individuals older than 65 years) [20]. Voluntary testing and testing of travelers were not covered by compulsory health insurance. Thus, other symptomatic COVID-19 cases seeking healthcare might have remained undiagnosed. Besides, PCR testing could have been unreliable due to untimely or incorrect specimen collection and limited viral replication in the epithelial cells of the upper respiratory tract [21, 22]. Therefore, recorded COVID-19 cases represented a subset of the actual number of the infected. Little is known about the spectrum of COVID-19 cases and the proportion of those who remained asymptomatic.

The World Health Organization recommended collecting self-reported COVID-19 history and performing a serological survey in a sample of the general population to support the retrospective assessment of the spread of the COVID-19 pandemic through the population [13].

Linkage of the data generated by different surveillance activities can lend insight into the spectrum of COVID-19 cases. The data accumulated in the case registry were described as providing accurate and reliable information on COVID-19 status in those captured by the healthcare system [12, 18]. In addition, self-reported survey data can provide a better capture of symptomatic cases, including those who did not seek medical advice [13]. A serological survey is used to detect those who were previously infected regardless of their symptomatic status and to assess the scale of the infection spread [23].

A previous population-based study in Arkhangelsk, Northwest Russia, conducted a year after the start of the pandemic, revealed a SARS-CoV-2 seroprevalence of 65.1% 95% CI (62.5; 67.6), with associations found between seropositive status and regular employment as well as smoking [24]. The current study combined the serological survey results, the healthcare data on the recorded cases (COVID-19 case registry), and the self-reported survey data on COVID-19 experience and symptoms. The study **aimed** to assess and describe the spectrum of COVID-19 cases in the sample of adult population a year after the start of the pandemic.

## Materials and methods

### Study population

The study was a satellite of a national multi-center survey of the prevalence of cardiovascular risk factors among adults aged 35–74 years in Russian regions (ESSE-RF3). The Arkhangelsk part of ESSE-RF3 was conducted by the Northern State Medical University (NSMU) between February 24, 2021 and June 30, 2021, a year after the start of the COVID-19 pandemic.

**Fig 1:**
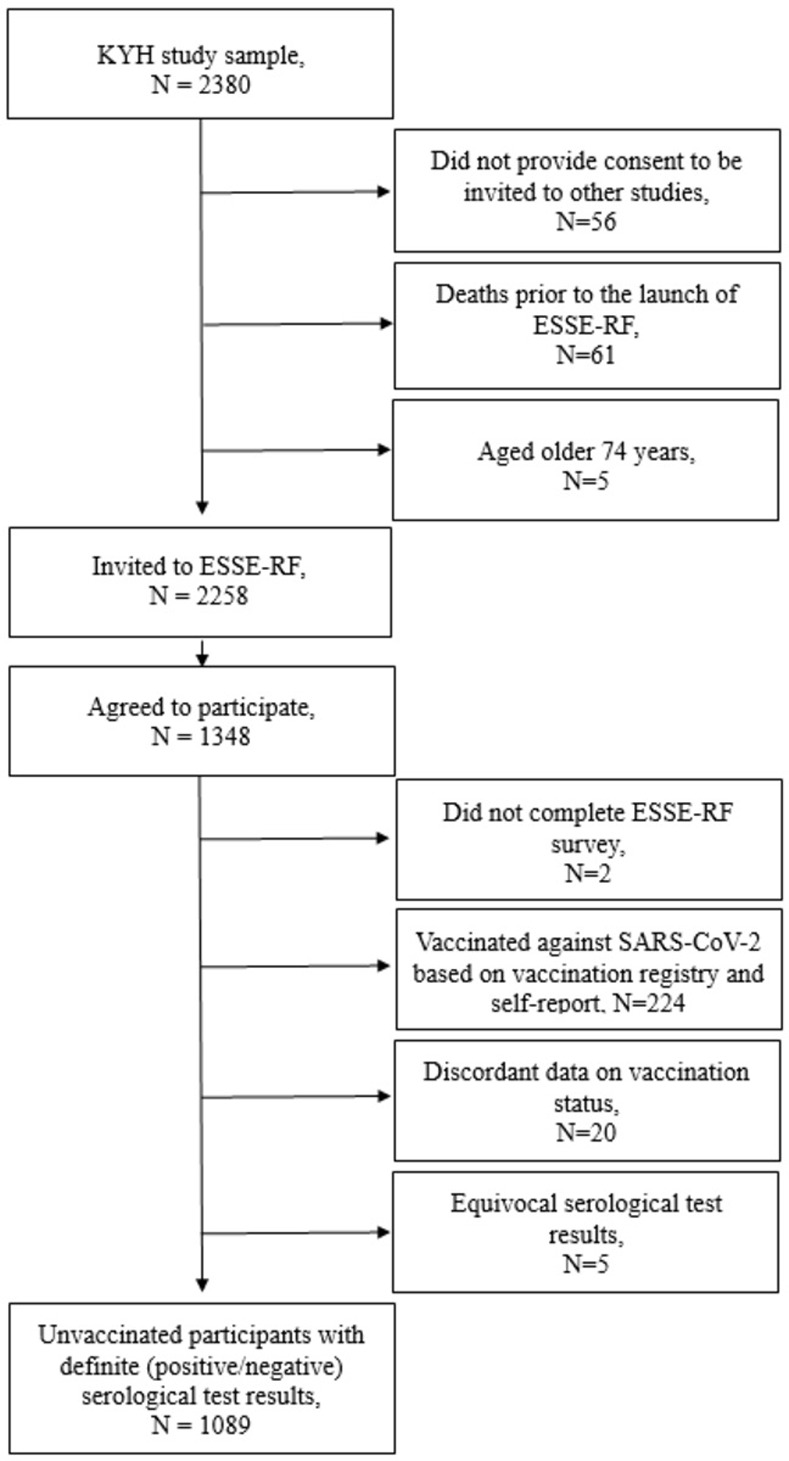
Flow chart of the study.

The ESSE-RF3 study sample in Arkhangelsk consisted of the participants of an earlier cross-sectional study of cardiovascular diseases ‒ Know Your Heart (KYH) [25]. The KYH study was conducted in 2015–2017 on a random sample (N = 2380) of Arkhangelsk population aged 35–69 years. The KYH participants were selected from four districts in Arkhangelsk using an anonymized list of addresses provided by the regional health insurance fund. Trained interviewers visited randomly chosen addresses, inviting individuals of the corresponding age and sex to participate. The overall participation rate was 68% among those invited. Based on the informed consent obtained from the KYH participants, they were invited to the ESSE-RF3 study by use of personal contact information. After the exclusion of those who had not consented to be contacted with new invitations (N = 56), those who had died prior to the launch of ESSE-RF3 (N = 61, including four deaths due to COVID-19), and those older than 74 years (N = 5) at the launch of ESSE-RF3 as exceeding the age span of the study, the list of invitees included 2258 KYH participants aged 40–74 years. With response rate of 59.7%, 1348 KYH participants attended the ESSE-RF3 study and comprised the current study sample. Two participants with incomplete ESSE-RF3 survey, 14 participants with equivocal serological test results, 224 individuals who self-reported receiving at least one dose of vaccine against SARS-CoV-2 and had a record in the vaccination registry, and 19 participants with discordant data on their vaccination status were excluded (Fig 1). Therefore, the analyzed study sample comprised 1089 unvaccinated participants with definitive serological test results.

### Ethics approval and consent to participate

Ethical approval for the KYH study was received from the ethics committees of the London School of Hygiene & Tropical Medicine (approval number 8808, February 24, 2015) and NSMU, Arkhangelsk (approval number 01/01-15, January 27, 2015). Ethical approval for the follow-up of the KYH using electronic health records was received from the ethics committee of NSMU, Arkhangelsk, Russia (approval number 01/04-19, April 24, 2019). Ethical approval for the ESSE-RF-3 was received from the ethics committee of the National Research Centre for Therapy and Preventive Medicine, Moscow, Russia (approval number 01-01/20, February 04, 2020) and the ethics committee of NSMU, Arkhangelsk, Russia (approval number 01/02-21, February 17, 2021). Ethical approval for the study of COVID-19-related issues was received from the ethics committees of NSMU, Arkhangelsk, Russia (approval number 01/02-21, February 17, 2021) and by the Regional Committee for Medical and Health Research Ethics (approval number 339397 received December 7, 2021).

All participants of the ESSE-RF study took part in the KYH study, and at the time of joining the KYH provided a written consent to disclose their medical and other health-related records for research purposes under the confidentiality condition and to be invited to other studies. The data linkage was performed by the Arkhangelsk Regional Medical Information Analytical Center (MIAC) in accordance with the NSMU-MIAC confidentiality agreement based on the informed consents obtained from the participants, as well as legal and ethical approvals. The dataset accessed and analyzed for this paper did not contain personal identifiers.

### Data collection

The data collection for ESSE-RF3 in Arkhangelsk involved a standardized interviewer-administered questionnaire survey, blood sample collection, and health examination. Trained interviewers collected data on participants’ demographic and lifestyle characteristics, self-reported diseases and vaccination. Trained physicians and nurses conducted health examination, including a medical interview (history and symptoms of diseases, medication use), blood sample taking, instrumental and functional measurements.

The following ESSE-RF3 variables were used in analyses: sex (male/female), age (5-year bands), higher education (yes/no), chronic health conditions (hypertension, diabetes, abdominal obesity), self-rated health, smoking, and frequency of heavy alcohol drinking. The participants with systolic blood pressure ≥ 140 mmHg and/or diastolic blood pressure ≥ 90 mmHg and/or using antihypertensive medications were treated as having hypertension. Diabetes was defined as self-reported diagnosis (including the type of diabetes) and/or self-reported use of antidiabetic medications and/or having glycated hemoglobin (HbA1c) ≥6.5%. Abdominal obesity was defined as waist circumference ≥94 cm for men and ≥80 cm for women. Self-rated health was measured on a 0–100 visual analogue scale orientated upwards (best) from below (worst) and then divided into two levels: lower or equal to median (poor health) and greater than median (good health). Smoking status was classified as non-smoker, former, and current smoker. Heavy alcohol drinking was defined as consuming of 60 or more grams of pure alcohol on a single occasion [26]. The frequency of heavy drinking during the previous 12 months was classified as never, once a week or less often, and twice a week or more often.

The history of prior COVID-19 was assessed by asking the participants “Did you have COVID-19 during the previous 12 months?” (yes/no/don’t know). We treated the “don’t know” response as a negative answer. Those who self-reported having had COVID-19 were asked to indicate the date of the disease onset and to answer the questions “Did you seek any medical advice?” and “Were you hospitalized?” with a yes/no answer options. They were also asked whether they experienced the following symptoms: fatigue, fever, headache, myalgia / arthralgia, loss of smell and taste (anosmia / ageusia), cough, dyspnea, sore throat, rhinitis, diarrhea, nausea / vomiting, and rash. We coded each symptom as 1 (present) or 0 (absent).

As the data were collected after the start of the COVID-19 vaccination campaign, the question “Have you received a vaccine for COVID-19?” was added to the ESSE-RF3 survey. Those responding positively were asked to provide information on the number of the doses (one or two) and the vaccination dates.

For the semi-quantitative detection of immunoglobulins G to spike glycoprotein of SARS-CoV-2, blood serum samples were analyzed using Vector Best enzyme linked immunosorbent assay (D-5501 SARS-CoV-2-IgG-EIA-BEST, Russia), a method with the 89% sensitivity and the 100% specificity reported by an independent test-performance study [27].

The self-reported survey data on prior COVID-19 and serological test results were linked with data from the COVID-19 case registry and the vaccination registry [17]. The following information was collected from the COVID-19 case registry: all COVID-19-related visits (outpatient and inpatient) prior to the participation in ESSE-RF3, including final diagnoses, date of the disease onset and the outcome. The study participants were treated as registered COVID-19 cases if they had records of COVID-19 diagnoses with codes U07.1 (COVID-19, virus identified) (N = 218, 96.5%) or U07.2 (COVID-19, virus not identified) (N = 8, 3.5%) according to the International Classification of Diseases, 10th revision. The code U07.2 was assigned to a clinical or epidemiological diagnosis of COVID-19 where laboratory confirmation was inconclusive or not available.

We used the vaccination registry to obtain the dates and the number of vaccination doses received by each vaccinated participant. The vaccination registry data were compared with the self-reported vaccination status. Self-reported vaccination details matched the vaccination registry records for 224 participants. Two participants self-reported no vaccination but had it recorded in the registry, and 18 self-reported vaccinations but had no corresponding records in the registry.

### Spectrum of COVID-19 cases

Based on the agreement between the serological test results, the COVID-19 case registry data and the self-reported survey data, the study participants were divided into those previously infected and non-infected (also referred to as ‘infected cases’ and ‘non-infected cases’ for short). The previously infected participants were classified as symptomatic or asymptomatic. Symptomatic cases were defined as having positive serological tests or records in the COVID-19 case registry and reporting COVID-19 symptoms at the survey. Asymptomatic cases were defined as having positive serological tests or COVID-19 record in the COVID-19 case registry, but reporting no symptoms of COVID-19 in the past. The participants having neither a positive serological test nor a record in the COVID-19 case registry were considered previously non-infected no matter what symptoms they reported. The infected cases were further grouped into non-hospitalized and hospitalized. The cases were defined as hospitalized if they were recorded as inpatients in the COVID-19 case registry (N = 53). Four participants who had had the infection and reported COVID-19 hospitalization with the dates and duration of the hospital stay, but had no records in the COVID-19 case registry were treated as hospitalized symptomatic cases.

### Statistical analysis

Absolute numbers and relative frequencies were presented for categorical data, and medians (first and third quartile) for continuous data. Confidence intervals (CIs) for proportions were calculated using Wilson’s procedure. We used the Pearson Chi-squared test to compare the frequency of non-infected, asymptomatic, non-hospitalized and hospitalized symptomatic cases among different groups of the participants. Binary logistic regression was used to examine factors associated with being a symptomatic COVID-19 case on a subset of infected cases (N = 650). Factors linked to hospitalization with COVID-19 were explored using binary logistic regression on a subset of symptomatic COVID-19 cases (N = 306). We employed directed acyclic graphs (DAGs) to justify the selection of covariates for adjustment (32). Findings were presented as crude odds ratios (ORs) with 95% CIs and ORs adjusted for demographic variables (age, sex, higher education) and lifestyle characteristics (smoking, frequency of heavy drinking), all selected from DAGs as potential confounders for all covariates (later in this paper referred to as the demographics and lifestyle factors). Adjusted ORs for smoking status were calculated comparing former smokers versus never-smokers, current smokers versus never-smokers, and ever-smokers (i.e., current and former smokers combined) versus never-smokers. Since there were no hospitalized patients among current smokers, the odds of hospitalization based on smoking status were estimated for former smokers compared with non-smokers. The threshold for significance tests was 0.05. The Statistical Package for the Social Science SPSS version 24.0 (SPSS Inc, Chicago, Il) was used for the data analysis.

## Results

The median age of the study sample was 55 (48; 63) years, 61.1% were women.

In total, 645 (59.2%) tested positive for immunoglobulins G to SARS-CoV-2, 220 (20.2%) had the infection documented in the case registry, and 316 (29.0%) self-reported COVID-19 symptoms.

**Fig 2:**
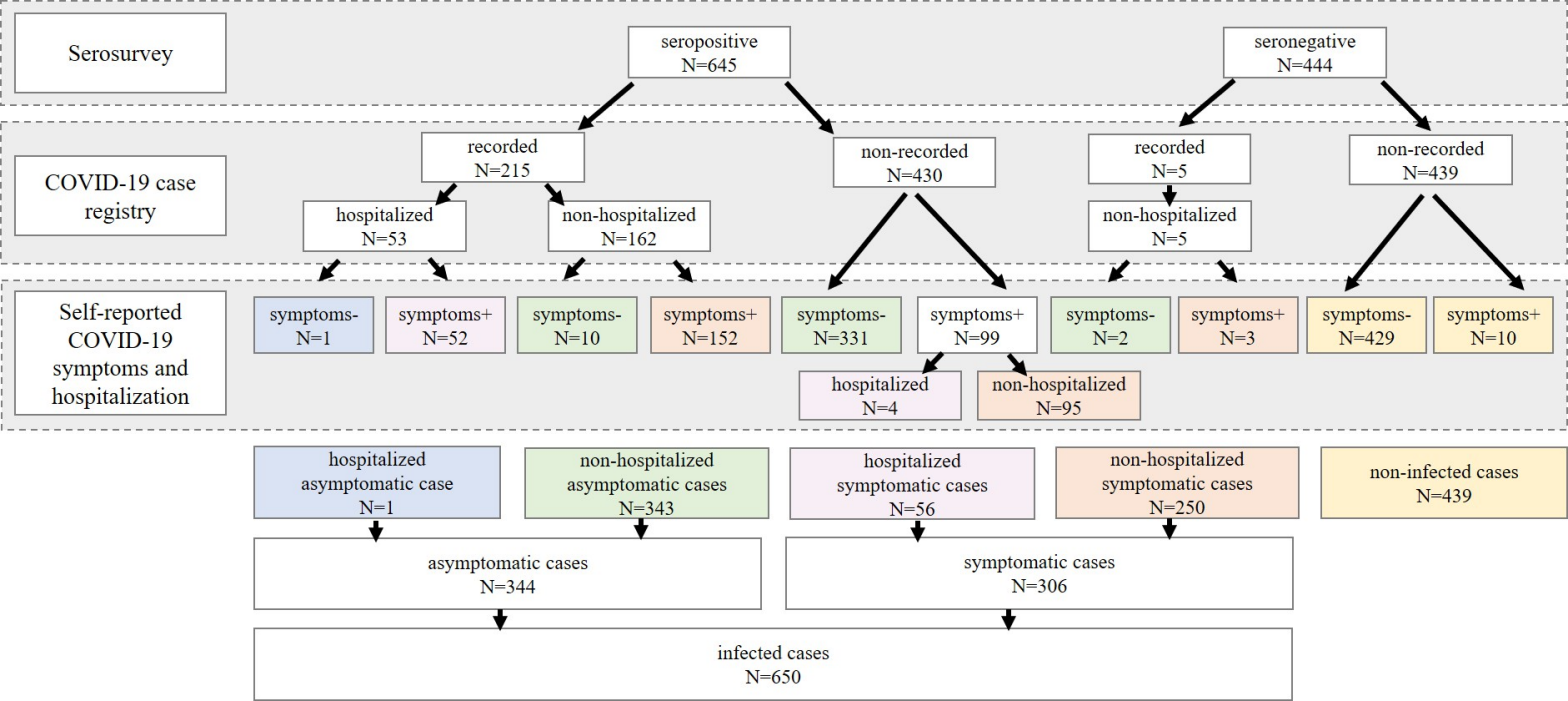
Classification of the study participants based on the linkage of the serosurvey, the COVID-19 case registry data and the self-reported survey data, N = 1089.

According to the linkage of case data from the three sources, the proportion of the participants who had had the infection in the studied sample was 59.7% 95% CI (56.7; 62.6) (N = 650). Asymptomatic COVID-19 cases comprised 52.9% 95% CI (49.0; 56.8) (N = 344) of all the infected (31.6% 95% CI (28.9; 34.5) of the study population). Of them, 96.2% (N = 331) were not captured by the case registry. The detailed information on the grouping of the participants is shown in Fig 2.

Less than half (47.1%, 95% CI (43.2; 51.0), N = 306) of the participants who had had the infection reported having had symptoms. Of them, 18.3% (N = 56) had been admitted to hospital (8.6% 95% CI (6.6; 11.1) of all previously infected).

**Fig 3:**
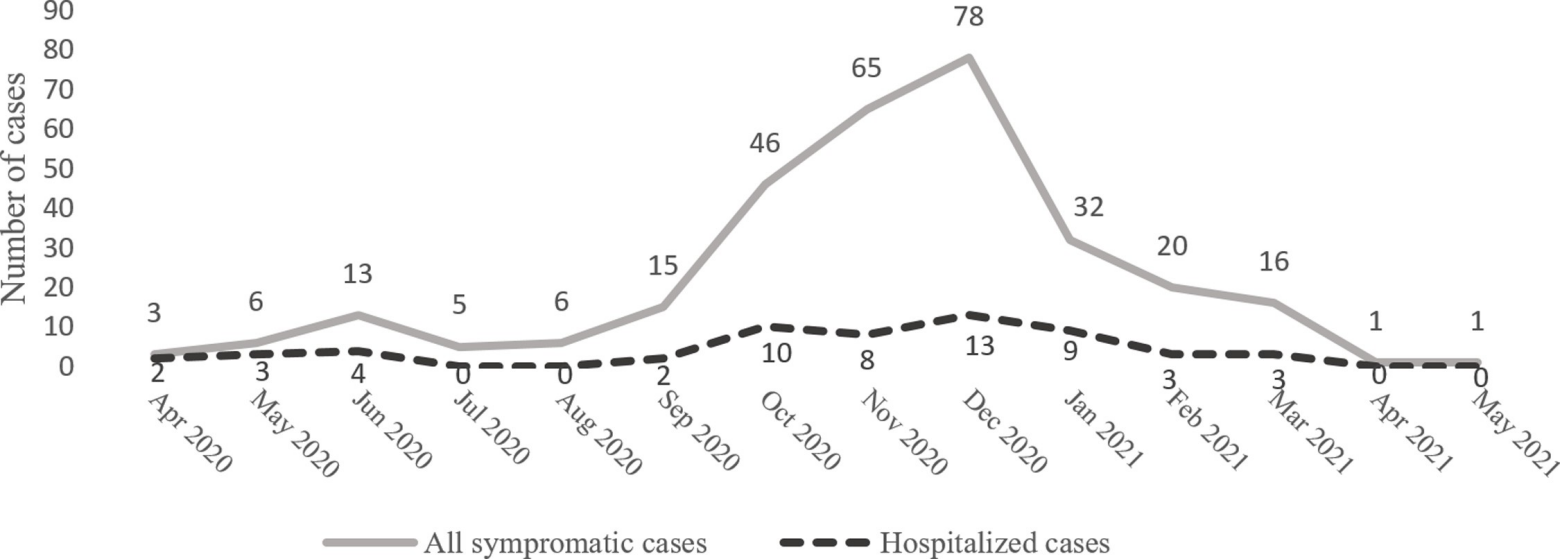
Distribution of the symptomatic cases over time.

The majority of the symptomatic cases (88.6%, N = 272) occurred between September 2020 and March 2021 with a peak in December 2020 (Fig 3). Almost a third of these cases (32.2%, N = 99) had no COVID-19 record in the case registry, despite the fact that most of them (88.9%, N = 88) had sought medical advice according to the survey data. Four of the non-recorded symptomatic cases (4.0%) self-reported having been hospitalized and indicated the date of the disease onset and the duration of the hospital stay.

The number of symptoms reported by the symptomatic cases (N = 306) varied from 1 to 12 with the median of 6 (5; 7). The most prevalent symptoms were fatigue (87.0%), fever (84.7%), headache (66.1%), myalgia / arthralgia (63.8%), anosmia / ageusia (63.5%), cough (51.1%), rhinitis (43.6%), dyspnea (41.7%), and sore throat (41.7%). Other symptoms included diarrhea (19.2%), nausea / vomiting (12.7%), and rash (3.3%). Most of the participants self-reported three or more symptoms (N = 285, 92.8%), and the remaining 7.2% were oligosymptomatic.

**Fig 4:**
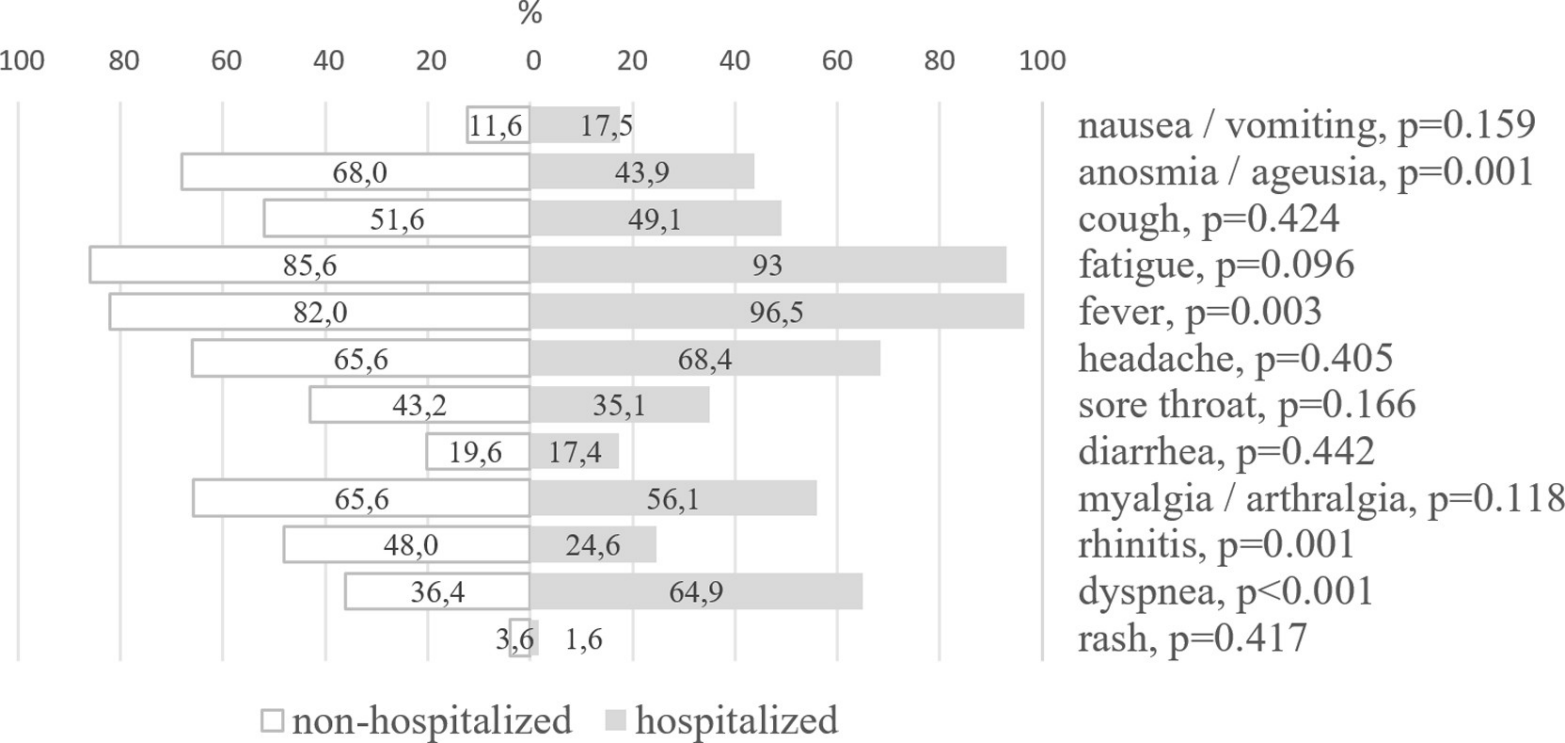
Symptoms self-reported by the non-hospitalized and hospitalized symptomatic patients, N = 306. p for Pearson’s Chi-squared test.

The median number of self-reported symptoms was similar for the hospitalized and non-hospitalized symptomatic cases. Compared with the non-hospitalized patients, the hospitalized cases had higher frequencies of fever and dyspnea. Anosmia / ageusia and rhinitis were more often reported by the non-hospitalized symptomatic COVID-19 cases compared to the hospitalized ones (Fig 4).

Table 1 presents the demographics, lifestyle, and health characteristics of non-infected, asymptomatic and symptomatic COVID-19 cases. The proportions of both asymptomatic cases and hospitalized symptomatic cases were higher in men compared to women (Table 1). The proportion of infected cases was higher in younger age groups. Among infected individuals, the frequency of symptomatic cases as well as the percentage of hospitalizations increased with age. Symptomatic participants with poor self-rated health, and those with diabetes had a higher proportion of hospitalizations. Current smokers and individuals with heavy drinking habits were less likely infected and less likely symptomatic cases. A quarter of former smokers had a symptomatic infection, with 7.3% being admitted to the hospital, while no current smokers were found among the hospitalized cases.

**Table 1:** Characteristics of non-infected and previously infected (asymptomatic and symptomatic COVID-19 cases).

Variable	Total N	Non-infected cases, N (%)	Asymptomatic cases, N (%)	Symptomatic non-hospitalized, N (%)	Symptomatic hospitalized cases, N (%)	p-value*
Sex						
Men	424	174 (41.0)	147 (34.6)	77 (18.1)	26 (6.3)	0.015
Women	665	265 (39.9)	197 (29.6)	173 (26.0)	30 (4.5)	
Age						
40–44 years	138	54 (39.1)	56 (40.6)	26 (18.8)	2 (1.5)	<0.001
45–54 years	386	132 (34.2)	142 (36.8)	102 (26.4)	10 (2.6)	
55–64 years	356	157 (44.1)	101 (28.4)	79 (22.2)	19 (5.3)	
65–74 years	209	96 (45.9)	45 (21.5)	43 (20.6)	25 (12.0)	
Higher education						
No	620	251 (40.5)	203 (32.7)	129 (20.8)	37 (6.0)	0.143
Yes	469	188 (40.0)	141 (30.1)	121 (25.8)	19 (4.1)	
Hypertension						
No	487	179 (36.8)	169 (34.7)	116 (23.8)	23 (4.7)	0.109
Yes	602	260 (43.2)	175 (29.1)	134 (22.2)	33 (5.5)	
Diabetes						
No	952	384 (40.3)	305 (32.0)	222 (23.4)	41 (4.3)	0.011
Yes	137	55 (40.1)	39 (28.5)	28 (20.4)	15 (11.0)	
Abdominal obesity						
No	476	189 (39.7)	153 (32.2)	112 (23.5)	22 (4.6)	0.867
Yes	613	250 (40.8)	191 (31.2)	138 (22.5)	34 (5.5)	
Self-rated health						
0–50%	212	88 (41.5)	64 (30.2)	39 (18.4)	21 (9.9)	0.003
51–100%	877	351 (40.0)	280 (31.9)	211 (24.1)	35 (4.0)	
Smoking status						
Non-smoker	621	226 (36.4)	196 (31.6)	164 (26.4)	35 (5.6)	<0.001
Former smoker	287	111 (38.7)	91 (31.7)	64 (22.3)	21 (7.3)	
Current smoker	181	102 (56.4)	57 (31.5)	22 (12.1)	0 (0.0)	
Frequency of heavy drinking						
Never	663	262 (39.5)	196 (29.6)	169 (25.5)	36 (5.4)	0.033
Once a week or less often	379	152 (40.1)	134 (35.4)	77 (20.3)	16 (4.2)	
Twice a week or more often	47	25 (53.2)	14 (29.8)	4 (8.5)	4 (8.5)	
Total	1089	439	344	250	56	

*p for Pearson’s Chi-squared test

Male sex, current smoking, and frequency of heavy drinking (once a week or less often) showed negative associations with symptomatic COVID-19 in the unadjusted models (Table 2). Older participants were more likely to be symptomatic COVID-19 cases. After adjustment for demographic and lifestyle factors, age was positively associated and smoking was negatively associated with symptomatic COVID-19. Adjusted estimates for ever-smokers (i.e. current and former smokers combined) versus never-smokers were 0.95 (95% CI 0.66;1.36) for symptomatic status.

**Table 2:** Factors associated with being symptomatic COVID-19 cases, N = 650.

Variable	OR crude (95% CI)	OR adj. (95% CI)^1^
Sex		
Women	reference	reference
Men	0.68 (0.49; 0.94)	0.74 (0.50; 1.08)
Age		
40–44 years	reference	reference
45–54 years	1.58 (0.94; 2.65)	1.56 (0.92; 2.64)
55–64 years	1.94 (1.14; 3.30)	1.93 (1.10; 3.36)
65–74 years	3.02 (1.68; 5.45)	2.96 (1.58; 5.53)
Higher education		
No	reference	reference
Yes	1.21 (0.89; 1.66)	1.30 (0.93; 1.81)
Hypertension		
No	reference	reference
Yes	1.16 (0.85; 1.58)	1.14 (0.81; 1.59)
Diabetes		
No	reference	reference
Yes	1.28 (0.80; 2.03)	1.10 (0.68; 1.78)
Abdominal obesity		
No	reference	reference
Yes	1.03 (0.75; 1.40)	0.92 (0.66; 1.28)
Self-rated health		
0–50%	1.07 (0.72; 1.58)	0.92 (0.61; 1.40)
51–100%	reference	reference
Smoking status		
Non-smoker	reference	reference
Former smoker	0.92 (0.65; 1.31)	1.15 (0.78; 1.70)
Current smoker	0.38 (0.22; 0.65)	0.55 (0.31; 0.97)
Frequency of heavy drinking		
Never	reference	reference
Once a week or less often	0.66 (0.48; 0.92)	0.91 (0.62; 1.34)
Twice a week or more often	0.55 (0.22; 1.33)	0.88 (0.34; 2.32)

^1^adjusted for sex, age, higher education, smoking, frequency of heavy drinking

In univariate analyses, men, older participants, individuals with poor self-rated health, diabetes, and those with heavy drinking habits (two times a week or more) were more likely to be hospitalized (Table 3). After adjustment for the demographics and lifestyle factors, age older than 65 years and poor self-rated health were associated with hospitalization. Adjusted OR for ever-smokers (i.e. current and former smokers combined) versus never-smokers was 0.95 (95% CI 0.45;1.99) for hospitalization.

**Table 3:** Factors associated with being hospitalized symptomatic COVID-19 case, N = 306.

Variable	OR crude (95% CI)	OR adj. (95% CI)^1^
Sex		
Women	reference	reference
Men	1.95 (1.08; 3.51)	1.93 (0.88; 4.17)
Age		
40–44 years	reference	reference
45–54 years	1.28 (0.26; 6.18)	1.28 (0.26; 6.36)
55–64 years	3.13 (0.68; 14.34)	2.82 (0.60; 13.40)
65–74 years	7.56 (1.65; 34.57)	6.99 (1.45; 33.72)
Higher education		
No	reference	reference
Yes	0.55 (0.30; 1.00)	0.76 (0.38; 1.51)
Hypertension		
No	reference	reference
Yes	1.24 (0.69; 2.24)	0.66 (0.33; 1.32)
Diabetes		
No	reference	reference
Yes	2.90 (1.43; 5.90)	2.22 (0.99; 5.00)
Abdominal obesity		
No	reference	reference
Yes	1.25 (0.69; 2.27)	1.15 (0.59; 2.24)
Self-rated health		
0–50%	3.25 (0.71; 6.16)	2.51 (1.23; 5.14)
51–100%	reference	reference
Smoking status		
Non-smoker	reference	reference
Former smoker	1.54 (0.83; 2.84)	1.25 (0.58; 2.66)
Current smoker	-	-
Frequency of heavy drinking		
Never	reference	reference
Once a week or less often	0.97 (0.51; 1.96)	1.03 (0.46; 2.27)
Twice a week or more often	4.69 (1.12; 19.65)	3.56 (0.63; 20.18)

^1^adjusted for sex, age, higher education, smoking, frequency of heavy drinking

## Discussion

In this study, we presented the spectrum and characteristics of COVID-19 cases in a sample of a population aged 40–74 in Arkhangelsk, Northwest Russia, a year after the start of the pandemic. The proportion of the participants who had had the infection was 59.7% 95% CI (56.7; 62.6) (N = 650). Of them, 47.1% 95% CI (43.2; 51.0) (N = 306) developed symptoms. Almost all of the symptomatic cases (96.4%, N = 295) had sought medical advice and 56 (18.3%) of them had been admitted to hospital. More than half (52.9% 95% CI (49.0; 56.8)) of the participants who had had the infection had been asymptomatic. Almost all of the asymptomatic cases (96.2%) had been undiagnosed and had not been included in the COVID-19 case registry.

The first case of COVID-19 emerged in Arkhangelsk on 17 March 2020. During the following six months of the pandemic, the Wuhan strain of SARS-CoV-2 predominantly circulated in the city. By the end of the summer of 2020, the incidence of COVID-19 had declined and stabilized [23]. Consequently, the local governmental restrictions, implemented on March 18, 2020, were relaxed starting from September 2020. Educational institutions resumed offline activities, and commercial and recreational activities were reinstated with the mandatory enforcement of a 1.5-meter distance rule, face mask wearing, hand antiseptic use, and non-contact thermometry [28]. The relaxation of restrictive measures coincided with the emergence of the Delta variant of SARS-CoV-2, with the first COVID-19 cases caused by the Delta variant registered in August 2020 [29]. Our findings show that the easing of restrictions, coupled with the Delta variant, boosted transmission (Fig 3), which is in line with another study conducted in Saint Petersburg, Northwest Russia [30]. It is likely that the proportion of infected cases in Arkhangelsk was higher compared to other Russian cities at approximately the same time [30, 31]. However, the differences could be attributed to the non-uniform sampling approaches used in the studies and the related variations in the characteristics of the studied samples [32].

In our study, 18.3% of the symptomatic cases (8.6% 95% CI (6.6; 11.1) of all previously infected) had been hospitalized. These findings are higher than those of Menachemi et al., who reported the hospitalization rate in the USA varied from 0.4% for those younger than 40 years to 9.2% for those older than 60 years [33]. In France, the proportion of the infected who needed hospitalization for COVID-19 from March 2020 to January 2021 was estimated to be 0.4% in individuals aged 20–29 years to 17.6% in those aged 70–89 years [34]. The high proportion of the participants who had been treated in hospital in our study could be partly explained by the common recommendation at early stages of the pandemic in Russia to isolate all detected COVID-19 patients by admitting them to hospital [20]. The percentage of hospitalized COVID-19 cases varies significantly across studies, depending on hospital admission policies in different countries at different time periods after the start of the pandemic and characteristics of the studied populations, such as age and comorbidities [20]. These inconsistencies make it difficult to compare the results of these studies.

In our study, the proportion of the hospitalized cases was higher in men compared to women, which is in line with other studies [35–38]. However, the association between sex and hospitalization did not persist after adjusting for age, higher education, smoking, frequency of heavy drinking. This suggests that the difference in hospitalization between men and women could be attributed to the varying prevalence of these factors between the sexes. Older age is a well-established factor associated with the disease severity [39, 40]. In support of this, we found a higher proportion of symptomatic cases and hospitalized cases among older study participants. The symptomatic COVID-19 patients with poor self-rated health were more likely to be hospitalized compared to those with good self-rated health. Our findings are in agreement with the results obtained by others, which demonstrated that comorbidities augmented the COVID-19 severity [41–43].

The highly specific COVID-19 symptom such as loss of smell and taste was more frequently reported by the non-hospitalized symptomatic cases compared to the hospitalized cases. Another study also found a higher frequency of the loss of smell and taste among individuals with mild COVID-19 [44], while other authors reported a positive correlation between these symptoms and COVID-19 severity [45]. Fever and dyspnea were more common in the hospitalized cases, which is in line with other studies [46, 47].

The proportion of participants who remained non-infected a year after the start of the pandemic was higher among older age groups, possibly due to non-pharmaceutical interventions, primarily self-isolation, specifically targeted at older individuals and those with chronic diseases [11, 28]. This may have contributed to a potentially lower overall severity of symptomatic COVID-19.

The proportion of the asymptomatic cases (52.9% 95% CI (49.0; 56.8) among all the infected exceeded the overall estimates published in the recent systematic reviews (40.5–44.1%) [48, 49]. Nevertheless, these estimates are influenced by the age groups included and should not be directly compared [32].

Limited testing access during the first months of the pandemic may have resulted in undiagnosed mild symptomatic infections among participants. Unaware of their COVID-19 status, these individuals might have erroneously reported having had no COVID-19 symptoms, potentially leading to misclassification as asymptomatic cases. The study design lacks symptom-related questions for those answering "No" to "Did you have COVID-19 in the previous 12 months?", possibly resulting to an underestimation of the number of symptomatic COVID-19 cases.

Although COVID-19 symptoms are non-specific, and incorporating questions about previous cold-like symptoms would have relatively low specificity, even with sensitivity close to 100% [50]. Many of these additionally revealed symptomatic cases would be false positives, falling into the category of non-hospitalized cases, resulting in differential misclassification.

Our findings are in line with earlier reports of a lower probability of symptomatic cases in younger age groups [14, 51]. Men were more prone to self-report being symptom-free, which is consistent with a number of studies [52, 53]. The associations between symptomatic status and sex disappeared after adjusting for age, education, smoking and frequency of heavy drinking. Thus, other factors, such as the varying prevalence of higher education, smoking and heavy drinking between the sexes, could contribute to the observed difference in symptomatic status. Like in other publications, we found a lower proportion of current smokers among symptomatic cases [54–56]. Current smokers had half the odds of being symptomatic COVID-19 cases compared to non-smokers, with none of the current smokers being hospitalized. Former smokers and ever-smokers (i.e. current and former smokers combined) had similar odds of being symptomatic COVID-19 cases as well as being hospitalized compared to non-smokers, which contradicts recent meta-analyses showing higher odds of severity and hospitalization among former and ever-smokers [57]. Former smokers may have quit smoking before or during the pandemic due to chronic diseases known to be risk factors for severe infection. Consequently, the overall health status of former smokers may be worse compared to that of current smokers [57].

The cross-sectional study design, along with possible biases, may influence the observed associations between smoking and disease severity. Simultaneous measurement of exposure and outcome may result in reverse causality. However, it is unlikely that adults aged 40–74 years initiated smoking after contracting COVID-19 [58]. Additionally, the possibility of reverse causality has not been suggested by other reports. We classified the study participants as non-smokers, former smokers, and current smokers. A more precise measurement of smoking status, including duration (years) or frequency (number of cigarettes) of smoking, may shed light on the studied association. As participant classification relied on self-reported smoking status, it could be influenced by bias related to the desire to undervalue socially unwelcome behaviors (misclassification) [59, 60]. Current smokers might be less prone to self-report symptoms and seek healthcare less frequently [61]. Consequently, they may be less likely to be tested for SARS-CoV-2 and to be captured in the COVID-19 case registry. Higher frequency of false-negative PCR tests in smokers may have contributed to the low prevalence of smokers among patients with COVID-19 [59, 62]. These factors could lead to an underrepresentation of current smokers among symptomatic COVID-19 cases.

As most studies [63–65] report a positive association between smoking and COVID-19 severity, the low frequency of symptomatic and hospitalized cases among current smokers in our study may also be explained by the possibility that they experienced severe illness and died before the initiation of the current study or refused to participate due to poor health conditions or post-COVID symptoms. The assumptions that tobacco smoke compounds reduce susceptibility to SARS-CoV-2 are speculative [66–68]. Further studies are required to properly explore how smoking status affects the course of COVID-19.

On the contrary to other studies, we found a lower proportion of symptomatic cases among those who reported frequent heavy drinking [35, 55]. However, after adjusting for age, sex, higher education, and smoking, frequency of heavy drinking was no longer associated with symptomatic status. At the same time, it should be noted that that regular alcohol drinking as well as smoking may decrease the probability of seeking medical advice or reporting symptoms among adults who experienced a disease or physical discomfort, as was shown in the previous study [61].

To our knowledge, this is the first population-based study in Russia estimating the COVID-19 spectrum. Combining COVID-19-related data from different sources enabled us to make reliable estimates of the proportion of the infected cases as well as the proportions of asymptomatic and symptomatic COVID-19 cases. However, our findings should be interpreted in the light of some limitations.

First of all, the sample of the current study was a resurveyed subsample of the previous population-based KYH study aged 40–74 years and may not be fully representative of the target population of Arkhangelsk residents of the same age. The response rate of 59.7% among the invited KYH participants could be a source of a non-response bias. To address this issue, we compared the participants of the current study to the entire KYH-based sampling frame on the key demographic variables (sex, age, education) [69]. Compared to the original KYH sample, resurvey participants had similar proportions of men and were younger on average at the time of inclusion in the KYH study. This is because the current study sample did not include participants of older age who died between the studies or could not participate in the current study due to severe illnesses. A slightly higher proportion of participants in the resurvey had higher education, possibly due to older participants who died or dropped out having a lower proportion of individuals with higher education. Some individuals may have attained higher education between the studies, and those with higher education may have increased health awareness and willingness to engage in research. These differences between the current study sample and the original KYH sample were unlikely to significantly impact the main results and conclusions.

We used the cross-sectional study design and therefore were not able to explore causal associations. Information bias could also impact the observed associations. Using data from multiple sources requires consideration of possible limitations and imperfect completeness of each of the sources used. For instance, our study results could be influenced by the registry data completeness and reliability [70]. Yet, we discovered only four cases showing discrepancy between the self-reported and the COVID-19 case registry data on hospitalization, which indicates only a negligible coverage deficiency of the registry with respect to hospitalized cases. Retrospective self-reported responses about symptoms may have been subjected to recall bias and non-differential misclassification of symptomatic status due to the difficulties of distinguishing between COVID-19 and other respiratory tract infections [71]. Those who had had COVID-19 but had not been tested might misclassify themselves as non-infected.

The number of the infected cases could have been underestimated due to the imperfect performance of the tests used as well as the antibodies waning over time after the infection [23]. To address this, the SARS-CoV-2 seroprevalence estimates described in our previous paper were adjusted for the serological test performance, and were increased by 7.9% compared to non-adjusted estimates [24]. Adjustments for test performance were not made in this study because the calculation of proportions of infected individuals considered data from various sources beyond the results of serological tests. However, the proportion of the infected cases and the proportion of the seropositive participants were almost the same in our study, with only five COVID-19 cases recorded in the case registry but tested negative for the SARS-CoV-2 antibodies. Thus, the underestimation might only be very small and could be due to the imperfect test sensitivity.

Alternately, we could overestimate the number of the seropositive people based on their cross-reactive immunity acquired after the infection caused by beta-coronaviruses with the molecular structure similar to SARS-CoV-2 [23, 72]. However, even though cross-reactivity was a concern early in the pandemic, there is limited evidence available. The standard procedure for validating the specificity of tests involves using pre-pandemic serum samples to ensure that the test accurately identifies true positives related to the virus and minimizes the likelihood of false positives caused by cross-reactivity [27]. Besides, some factors that could not be assessed in our study, such as the subtype of SARS-CoV-2 or the viral load, may influence the symptomatic status [73].

The study added to the knowledge of the COVID-19 spectrum in a Russian sample with a high proportion of the previously infected, but it may be of a limited novelty with respect to symptomatic COVID-19 cases. The proportion of the people who had been infected and remained asymptomatic was a key question in understanding the extent of COVID-19 in the study setting. Estimating the proportion of asymptomatic COVID-19 cases is critical for calculating key epidemiological characteristics, quantifying the cumulative incidence of the infection. An exact evaluation of the proportion and a better understanding of characteristics of asymptomatic cases could help to retrospectively assess the strategies which were implemented to control the COVID-19 pandemic. The identification of a considerable number of undetected cases underscores the potential lessons to be learned from the existing surveillance and contact tracking strategy, suggesting areas for improvement in infection control measures. Several studies have shown that asymptomatic individuals shed similar quantities of virus to the symptomatic persons [14, 15]. Thus, being unaware of their disease status, the asymptomatic individuals could unknowingly transmit the virus to others [74].

## Conclusion

A year after the start of the COVID-19 pandemic in Arkhangelsk, Northwest Russia, 59.7% 95% CI (56.7; 62.6) of the surveyed adult population aged 40–74 years had been infected by SARS-CoV-2. Symptomatic cases comprised 47.1% 95% CI (43.2; 51.0) of the total infected, and 8.6% 95% CI (6.6; 11.1) of those previously infected were hospitalized. Our findings indicated a high proportion of asymptomatic cases that remained undetected by the healthcare system. The asymptomatic COVID-19 cases were unaware that they had been infected and might have continued their usual activities spreading the infection to others. This could have resulted in the rapid COVID-19 transmission and unsuccessful disease control.

Since the asymptomatic COVID-19 patients are difficult to be diagnosed, a wider testing of high-risk populations should be performed regardless of symptoms to improve the control strategies. Combining different surveillance approaches could prevent future outbreaks by capturing silent infection spread.
